# The Effect of Group Support Psychotherapy Delivered by Trained Lay Health Workers for Depression Treatment Among People with HIV in Uganda: Protocol of a Pragmatic, Cluster Randomized Trial

**DOI:** 10.2196/resprot.8925

**Published:** 2017-12-11

**Authors:** Etheldreda Nakimuli-Mpungu, Seggane Musisi, Kizito Wamala, James Okello, Sheila Ndyanabangi, Ramin Mojtabai, Jean Nachega, Ofir Harari, Edward Mills

**Affiliations:** ^1^ Department of Psychiatry College of Health Sciences Makerere University Kampala Uganda; ^2^ Department of Psychology Center for Victims of Torture Gulu Uganda; ^3^ Department of Mental Health Faculty of Medicine Gulu University Gulu Uganda; ^4^ Mental Health Program Ministry of Health of Uganda Kampala Uganda; ^5^ Department of Mental Health Bloomberg's School of Public Health Johns Hopkins University Baltimore, MD United States; ^6^ Department of Epidemiology Pittsburg Graduate School of Public Health University of Pittsburg Pittsburgh, PA United States; ^7^ Stellenbosch Center for Infectious Disease Department of Medicine Stellenbosch University Cape Town South Africa; ^8^ Department of Epidemiology Bloomberg's School of Public Health Johns Hopkins University Baltimore, MD United States; ^9^ Department of International Health Bloomberg's School of Public Health Johns Hopkins University Baltimore, MD United States; ^10^ MTEK Sciences Inc Vancouver, BC Canada; ^11^ Department of Clinical Epidemiology & Biostatistics McMaster University Hamilton, ON Canada

**Keywords:** cluster randomized trial, group support psychotherapy, lay health workers, depression, persons living with HIV/AIDS, Uganda

## Abstract

**Background:**

There is limited information on the effectiveness of task shifting of mental health services in populations with HIV.

**Objective:**

This trial aims to evaluate the effectiveness of group support psychotherapy delivered by trained lay health workers to persons living with HIV (PLWH) with depression in primary care.

**Methods:**

Thirty eligible primary care health centers across three districts were randomly allocated to have their lay health workers trained to deliver group support psychotherapy (intervention arm) or group HIV education and treatment as usual (control arm) to PLWH with depression. Treated PLWH will be evaluated at baseline, after the end of treatment, and at 6-month intervals thereafter for 2 years. Primary outcomes will be the difference in follow-up proportions of participants with Mini International Neuropsychiatric Interview criteria for major depression and difference in follow-up function scores of participants in the intervention and control arms 6 months after the end of treatment. Secondary outcomes will include measures of self-esteem, posttraumatic stress symptoms, social support, stigma, adherence to antiretroviral therapy, viral load, and number of disability days, asset possession indices, and cost-effectiveness data. Primary and secondary outcomes as well as subgroup analyses will be conducted at the individual level using multilevel random effects regression analyses adjusting for clustering in health centers. A process evaluation using mixed methods to assess acceptability, feasibility, fidelity, causal mediating processes, and contextual influences in the trial will be conducted.

**Results:**

The trial has been approved by the Makerere College of Health Sciences School of Health Sciences Research Ethics Committee, the AIDS Support Organization, and the Uganda National Council of Science and Technology. A data and safety monitoring board has been put in place to monitor trial progress. A total of 1140 persons living with HIV have been recruited to the trial. An analysis of baseline and 6-month data is in progress. The results of this trial will not only be presented at national and international conferences but also submitted for publication in peer-reviewed journals and as a report to the funding agencies.

**Conclusions:**

This cluster randomized trial will provide critical evidence to support culturally sensitive group-based psychotherapy for depression treatment in sub-Saharan Africa. Process evaluation outcomes will provide contextual information that health care and public health stakeholders can use to guide implementation decisions for their particular setting.

**Trial Registration:**

Pan African Clinical Trials Registry (PACTR): 201608001738234; http://www.pactr.org/ATMWeb/ appmanager/atm/atmregistry?dar=true&tNo=PACTR201608001738234 (Archived by WebCite at http://www.webcitation.org/ 6vUAgAQlj)

## Introduction

In the fight against the HIV epidemic over the past three decades, one of the major barriers to universal access to health care needed by persons living with HIV (PLWH) has been a serious shortage of health workers to deliver these interventions [1]. The shortage of well-trained health workers to address the myriad biological, social, and psychological challenges of living with HIV poses a risk for suboptimal HIV treatment outcomes, particularly in sub-Saharan African countries where the burden of HIV and acquired immune deficiency syndrome (AIDS) is greatest [2]. For example, the lack of capacity to provide mental health care such as screening for depression in HIV care programs results in undetected and untreated depression, which impairs the ability to adhere to antiretroviral (ART) medications [3,4]. Affected individuals continue to struggle with poor health, social, and economic outcomes because depression impairs their ability to function in their families, at work, and in their communities [5,6].

The World Health Organization (WHO) HIV treatment guidelines now recognize depression treatment as central to effective HIV treatment programs [7]. This, in turn, has resulted in the development and testing of a number of psychological interventions for depression that can be used in poor resource areas where the gap between the availability and need for mental health services [8-10], known as the “treatment gap,” may be as high as 90% [11]. Unfortunately, accessibility and sustainability of these interventions is impeded by the severe shortage of mental health professionals who can deliver them to affected individuals who need them [12].

Task-shifting approaches, whereby nonspecialist health workers in primary care and community settings are trained to deliver some of the mental health services that were provided solely by specialist mental health professionals, have been recommended by global mental health researchers and practitioners [13] as well as the WHO. The WHO recommends that these task-shifting approaches must use standardized and simplified interventions that can realistically be administered by less highly trained professional health care workers and nonprofessional community members [2]. However, studies describing the implementation of task-shifting approaches in delivery of psychological interventions for PLWH in sub-Saharan Africa are limited.

In a recent review of studies documenting the effectiveness of psychological interventions for PLWH in low- to middle-income countries, none of the studies that met criteria for inclusion in the review demonstrated the use of a task-shifting approach in the delivery of the intervention [14]. In a recent review of studies describing mental health training of health care workers in Africa, only three of the 37 studies reviewed described training of lay health workers (LHWs) [15]. There is a need for information on how a task-shifting approach can be used to make a psychological intervention for depression accessible and sustainable in low-resource settings.

The shifting of mental health‒related tasks from health professionals to LHWs has been repeatedly documented in non-HIV populations [16-19]. However, because mental health has not been integrated into HIV care in sub-Saharan Africa, little is known about the effectiveness of task shifting of mental health‒related services such as depression care. Also, identifying mediators and moderators of intervention response is a critical step in understanding the mediating causal factors as well as for whom and under which conditions an intervention is most beneficial [20]. This knowledge also has important implications for practice, as it can aid in tailoring and modifying interventions so as to be maximally effective for specific target populations.

To address this knowledge gap, we set out to conduct a cluster randomized trial to test the effectiveness of group support psychotherapy (GSP) delivered by trained LHWs. The recommended methodological approach to establish the efficacy of a newly developed therapy such as GSP is to first compare the therapy against a comparison group that omits the unique ingredients of the new therapy while possessing the common factors (eg, therapeutic alliance) in equal measure [21]. Thus, we compared the effects of GSP to those of group HIV education (GHE) delivered by trained LHWs on mild to moderate depression and functioning among PLWH attending primary care health centers in northern Uganda. We hypothesized that compared with the control arm, the proportion of participants that meets the Mini International Neuropsychiatric Interview (MINI) criteria for major depression will be lower and the function scores will be higher in the intervention arm 6 months after the end of treatment.

Secondarily, we aimed to compare the effects of GSP and GHE delivered by trained LHWs to PLWH presenting with mild to moderate depression in primary care on measures of self-esteem, posttraumatic stress symptoms, social support, stigma, adherence to ART, viral load, number of disability days, asset possession, poverty indices, and cost-effectiveness measures. We hypothesized that compared with PLWH receiving GHE, those receiving GSP will achieve a greater increase in social support and positive coping skills and greater reduction in stigma; larger increases in adherence to ART and greater reduction in viral load; and greater reduction in number of disability days, greater increase in asset possession scores, and larger reductions in poverty index scores at 6, 12, 18, and 24 months after the end of treatment.

We also plan to conduct a process evaluation of trial activities informed by Linnan and Steckler’s process evaluation frameworks [22] to specifically determine indicators of feasibility including *reach* (ie, the proportion of participants who received the intervention), *dose delivered and received* (ie, the amount of intervention delivered and the extent to which participants respond to it), as well as *attrition* (ie, the proportion of participants who are lost to follow-up); indicators of acceptability including satisfaction with intervention content, delivery agents and effects; *fidelity* (ie, whether the intervention is delivered as planned); and causal mediating processes and contextual influences. Given that gender has far-reaching implications for social roles, opportunities, and experience of adversities in traditional societies such as in Uganda [23], we will determine whether or not the effects of GSP are moderated by gender. Both posttraumatic stress [24] and alcohol use disorders [25] are associated with an increased risk for depression. Therefore, we will also examine whether or not the effects of GSP are moderated by psychiatric comorbidities. Extensive reviews of literature [26,27] have found that common factors such as the therapeutic relationship may account for up to nine times greater impact on patient improvement than the specific mechanisms of action found in formal treatment protocols. Therefore, we shall assess whether the strength of a therapeutic relationship will mediate the effects of GSP on depression and subsequently other study outcomes. Identifying groups of individuals for whom GSP works best will assist in the goal of developing selection criteria to guide the referral of patients for GSP.

## Methods

### Study Design

This is a pragmatic two-arm cluster randomized trial evaluating the effectiveness GSP delivered by trained LHWs to persons with HIV presenting with mild to moderate depression in primary care. The study involves LHWs affiliated to 30 primary care health centers (PHCs) in three districts in northern Uganda. Eligible PHCs were randomly assigned (1:1) to have their LHWs trained in the delivery of GSP (intervention) or GHE (control) to PLWH with mild to moderate depression. PLWH treated by trained LHWs will be evaluated at baseline, at the end of intervention, and at intervals of 6 months thereafter for 2 years.

A longitudinal process evaluation of the delivery of GSP by trained LHWs using mixed methods will run alongside the trial to assess acceptability, feasibility, fidelity, and how intervention recipients respond to the different intervention components. Analysis is performed according to the intention-to-treat (ITT) principle and will account for the cluster randomized design. The study protocol is registered in the Pan African Clinical Trials Registry (PACTR201608001738234), and the reporting of the trial will be in accordance with the Standard Protocol Items: Recommendations for Interventional Trials (SPIRIT) guidelines [28] for intervention trials (see Multimedia Appendix 1) and the CONSORT statements for cluster randomized trials [29].

**Figure 1:**
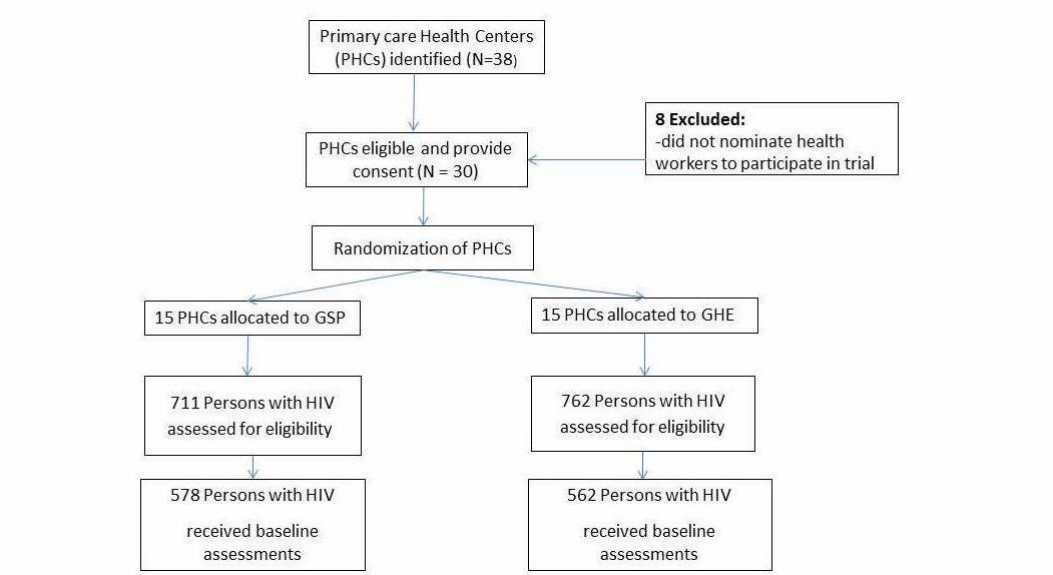
Trial flowchart: selection of clusters and participants.

The study was submitted to and approved by both the Makerere University College of Health Sciences Research Ethics Committee and the Uganda National Council of Science and Technology. All study participants will be required to provide written informed consent. Light refreshments will be served during all group sessions in both arms, and every participant will receive a financial incentive to defray transport costs. Figure 1 summarizes the trial profile.

### Study Setting

This trial has been implemented in 30 PHCs situated in three districts in northern Uganda (Gulu, Kitgum, and Pader) with a population of 450,000, 247,000, and 250,000, respectively. Over 90% of the population is engaged in small scale agriculture and animal husbandry as their major income-generating activity. The brutal civil war that the people of these districts endured for two decades (1987-2007) led to a breakdown of health care delivery systems, disorganized social and cultural life with minimal economic activity, and loss of property and infrastructure in these districts. This situation of precariousness led to high poverty rates, risky behaviors including excessive alcohol consumption, and sexual violence making the population highly vulnerable to depression and HIV/AIDS [30].

The first level of health care accessible to this study population is provided by LHWs who work in teams referred to as village health teams. The village health team is affiliated with other PHCs at the second level (Health Center II), third level (Health Center III), or fourth level (Health Center IV), which are led by professional health workers who supervise their outreach activities [31].

### Participants

#### Cluster Eligibility Criteria, Recruitment, and Randomization

To be eligible for the trial, the primary care health centers had to offer HIV care and treatment services, nominate at least four LHWs (2 men and 2 women) who are actively involved in HIV care, able to read and write, and reside within the villages served by the PHC. PHCs that do not offer HIV care services and those unable to nominate literate LHWs were excluded from the study.

To recruit PHCs, the project team worked with the district health officials in each of the three participating districts and identified a total of 38 PHCs that offered HIV care and treatment services. The health center managers of these centers were informed about the trial and asked to nominate health workers to participate in the trial. The first 10 PHCs in each district that expressed interest in the trial and nominated the required number of health workers were recruited to the trial. The health center managers of the 10 eligible PHCs in each district were invited to a stakeholders’ meeting held at the district’s local government offices with political leaders, district health officials, religious leaders, and community leaders. Study purpose and procedures were explained to facilitate district leadership understanding of the trial activities. At the end of the meeting, the eligible PHCs were randomly allocated with a 1:1 ratio to intervention and control arms. Randomization was done by urn randomization picked by each health center manager or their representative who attended the stakeholders’ meeting.

#### Participant Eligibility Criteria, Recruitment, and Masking

To be eligible for the study, PLWH had to be 19 years and older, diagnosed with major depression assessed with the MINI depression module [32], antidepressant naïve, using ART, and residing in the villages where the trained LHWs lived. Individuals with high suicide risk [33], a severe medical disorder such as pneumonia or active tuberculosis, psychotic symptoms, and hearing or visual impairment were excluded from the study.

On a given clinic day, project research assistants worked with primary care health workers of a participating PHC in the center’s HIV clinic. The primary care health worker gave a health talk on depression to clients in the waiting area. Clients who felt that they had experienced symptoms of depression described in the health talk were invited for further evaluations using the Luo version of the 20-item self-reporting questionnaire [34] and the MINI depression module. This procedure was repeated until a total of 40 PLWH diagnosed with major depression were obtained from a given PHC.

Clients diagnosed with major depression were approached by research assistants who explained study procedures, determined eligibility, and then obtained informed consent. Each client who gave informed consent received baseline assessments with a standardized questionnaire. Recruited participants from the same village were assigned to a trained LHW residing in or near their village to receive the intervention they had been trained to deliver (ie, either GSP or GHE).

By design, both experimental and control interventions were identifiable to participants but will be masked to outcome assessors, the Data and Safety Monitoring Board, and data analysts.

### Interventions

The development of the GSP and GHE interventions has been described in detail in previous publications [35,36]. Multimedia Appendix 2 shows a detailed description of the content of both interventions, which has also been previously published [37].

#### Group Support Psychotherapy Training

Over a 4-month period (January-April 2016), Makerere University in collaboration with the Ministry of Health designed a GSP training program that consists of both formal and informal training. Between May and August 2016, the training-of-trainers model was used to deliver the training, whereby mental health specialists trained PHC health workers who in turn trained the LHWs. Formal training consisted of 8 training modules delivered in a 5-day training workshop that employed active learning techniques including role plays, brainstorming sessions, and small group discussions.

In brief, the first three modules including an overview of the training program, introduction to the GSP model, and introduction to depression and HIV/AIDS were delivered on the first day. On the second and third days, modules on basic counseling skills and effective coping strategies were delivered, respectively. On the fourth day, participants received training in basic livelihood skills (enterprise selection, basic financial skills, and resource mobilization) required to overcome poverty. The last day of training focused on self-care strategies, post-training assessments, and training workshop evaluation. Informal training consisted of conducting supervised pilot GSP sessions. Multimedia Appendix 3 summarizes the competencies targeted by the training.

#### Group HIV Education Training

In May 2016, Makerere University in collaboration with The AIDS Support Organization (TASO), designed a group HIV education training program that consisted of both formal and informal training. Between May and August 2016, the training-of-trainers model was used to deliver the training, whereby TASO HIV care providers trained PHC health workers who in turn trained the LHWs. Formal training consisted of five training modules delivered in a lecture format in a 2-day training workshop. In brief, on the first day, three modules including an overview of the training program, introduction to depression and HIV/AIDS, HIV progression and transmission were delivered. On the second day, modules on mother-to-child transmission and basic facts on ART were delivered. Informal training consisted of conducting supervised pilot GHE sessions.

#### Intervention Fidelity

Between September and December 2016, trained LHWs delivered the interventions to PLWH with mild to moderate depression recruited to participate in the trial. Strategies to ensure treatment fidelity in both treatment arms included the use of standardized intervention materials, structured health worker training, ongoing supervision, and training a larger number of LHWs than was required in order to avoid potential disruptions due to illness or job transfers. The LHWs underwent standardized training by trained primary care health workers. Each LHW delivered GSP or GHE following a manual translated into the local language. Prior to the sessions, each LHW drew up a schedule indicating which day of the week each group session would take place and handed it over to their supervisor. Of the 8 sessions to be delivered, 3-4 sessions were supervised by a trained PHC worker who assessed the LHW’s basic counseling skills using a supervision checklist with items adapted from the Enhancing Assessment of Common Therapeutic Factors scale [38].

In addition, the LHWs who facilitated group sessions were required to complete a self-administered semistructured feedback questionnaire after each group session in which they described content delivered in each group session and provided feedback on facilitators and barriers faced during the group session. Their feedback was discussed with their supervisors who advised on how to address any difficult issues raised. From this feedback, supervisors were able to assess whether the intervention delivery progressed as planned or not and to intervene accordingly.

### Participant Safety

During baseline assessments, participants were carefully screened and individuals for whom the interventions were deemed medically inappropriate or unsafe were excluded. Outcome assessors screened all participants for adverse events at the end of treatment and at 6 months after the end of the intervention using a standard interview and reporting form. Going forward, follow-up assessments will continue at 6-month intervals up to 2 years after the end of treatment. Any unfavorable and unintended sign or symptom associated with the participation in either intervention, regardless of whether it is considered related to the therapy is reviewed by the investigating team for seriousness, study relatedness, and expectedness. Similar information reported by participants at other times (eg, during intervention encounters) is duly noted and followed up with, as needed, to assure participant safety. Adverse events are reported according to the data and safety monitoring plan shown in Multimedia Appendix 4.

### Retention

Currently, trial participants have received their 6-month outcome assessment and will be due for their 12-month outcome assessment in January-February 2018. In order to maximize adherence and retention, we formed community advisory boards to monitor community satisfaction with implementation activities and to provide feedback to investigators in real time on any conflicts or dissatisfaction arising from project implementation activities. Lay health workers who facilitated the group sessions and the primary care health workers who supervised them were provided with a financial incentive as an appreciation of their commitment to the project. For participants who missed group sessions, the LHWs made home visits to re-engage them. Given that group sessions took place in the villages, the LHWs know the homes of the participants and have been able to mobilize them to return for their follow-up assessments.

### Study Measures and Data Collection Schedule

So far, assessments of study measures have been conducted at baseline, at the end of the interventions (2 months), and 6 months after the interventions. Going forward, further assessments will be conducted at 12, 18, and 24 months after the intervention. Table 1 [32,34,39-47] summarizes the study measures including the primary and secondary outcomes, process evaluation outcomes, potential effect modifiers, and mediators.

**Table 1:** List of study measures and data collection schedule.

Study measures		Instrument	Data collection schedule (months)					
			0	2	6	12	18	24
**Primary outcomes**								
	Major depression	The Mini International Neuropsychiatric Interview [32]	✓		✓	✓	✓	✓
	Functioning level	5-item locally developed function assessment method [39]	✓		✓	✓	✓	✓
**Secondary outcomes**								
	Depression symptoms	Self-Reporting Questionnaire [34]	✓		✓	✓	✓	✓
	Posttraumatic stress symptoms	Locally adapted Harvard Trauma Questionnaire [40]	✓		✓	✓	✓	✓
	Alcohol use	10-item Alcohol Use Disorders Identification Test [44]	✓		✓	✓	✓	✓
	Disability days	Single question “How many working days have you lost due to depression-related symptoms in the previous 30 days?	✓		✓	✓	✓	✓
	Cost-effectiveness	Estimation of incremental costs of the GSP intervention arm as compared to the control arm	✓		✓			✓
	Poverty index scores	Questions on indicators of socioeconomic status such as the building materials of the household dwelling unit, access to electricity, type of cooking fuel, source of lighting, household remoteness (distance to nearest health facility), household food security, household durable assets such as radios, televisions, and mobile phones	✓		✓			✓
	Adherence to ART	One question: “During the past week, on how many days have you missed taking all your medication doses?”	✓		✓	✓	✓	✓
	Viral load	Medical charts of study participants	✓		✓		✓	
**Potential effect modifiers**								
	Sociodemographic variables	Standardized Demographic Questionnaire	✓					
	Trauma events	Locally developed 16-item trauma event checklist	✓					
**Potential effect mediators**								
	Self-esteem	Rosenberg Self-Esteem Scale [41]	✓		✓	✓	✓	✓
	Social support	12-item Multi-Dimensional Perceived Social Support scale [42]	✓		✓	✓	✓	✓
	HIV-related stigma	HIV-related stigma scale [43]	✓		✓	✓	✓	✓
	Coping skills	COPE Inventory [45]	✓		✓	✓	✓	✓
	The therapeutic relationship	The Scale to Assess the Therapeutic Relationships–Patient version [46]		✓				
**Process evaluation outcomes**								
	Indicators of feasibility	The proportion of eligible participants who take up either intervention (Reach), the proportion who attended all 8 sessions of either intervention (dose delivered), and the proportion who are lost to follow-up (attrition) will be determined from the attendance registers		✓				
	Indicators of acceptability	A 9-item questionnaire [47] will assess participant’s satisfaction, the group facilitators’ knowledge and attitudes, and the participant evaluation of the intervention’s ability to reduce depression.			✓			
	Fidelity	A semistructured self-administered questionnaire completed by group facilitators will assess whether or not the interventions were delivered as planned.		✓				
	Contextual influences	A semistructured self-administered questionnaire completed by group facilitators will assess any facilitators or barriers to intervention delivery that they observed during group sessions.			✓			
	Causal mechanisms	A semistructured interviewer-administered questionnaire will assess participant’s knowledge, skills, or assets acquired during and after the interventions, which will give an indication as to whether or not the interventions influenced targeted risk factors for depression.			✓			

**Table 2:** Sample size and power calculations.

Alpha	Beta	*k*	Cluster size	Control group proportion	Intervention group proportion	Minimum clusters required	Resulting power
.01	.2	.25	32	0.25	0.15	18	0.808
.05	.2	.25	32	0.25	0.15	12	0.803
.01	.2	.25	40	0.25	0.15	16	0.818
.05	.2	.25	40	0.25	0.15	11	0.823
.01	.2	.25	32	0.3	0.15	10	0.842
.05	.2	.25	32	0.3	0.15	7	0.849
.01	.2	.25	40	0.3	0.15	9	0.851
.05	.2	.25	40	0.3	0.15	6	0.84

### Sample Size and Power Calculations

Primarily, our objective is to compare the proportion of subjects with mild to moderate depression in the intervention and control arms 6 months after the end of treatment. Based on results from our pilot project, we assume that the difference in proportion of depression cases at 6 months follow-up between intervention (15%) and control arms (25%) would be 10%. Using formulae proposed by Hayes and Moulton [48], and assuming the between-cluster coefficient of variation *k* of .25, a study with 12 nonmatched pairs of clusters, and a cluster size of 32 PLWH (total expected sample size of 768 PLWH) would have 80% power of detecting a 10% reduction in major depression cases at the 5% significance level. Table 2 illustrates the sample size and power calculations for various assumptions made. The number of clusters has been increased to 15 pairs to allow for individual level analyses using multilevel random effects regression models, and the cluster size was increased to 40, accounting for a potential 20% loss to follow-up.

### Data Collection and Management

Study participants in the intervention and control arms will be asked to complete an interviewer-administered face-to-face standardized questionnaire to collect data on primary and secondary outcomes at baseline (T0), after completion of their group treatments (T1), and 6, 12, 18, and 24 months after end of treatment (T2, T3, T4, and T5 respectively). All the completed survey questionnaires will be reviewed by a research team member for missing data and unusual responses. Data will be entered into an EpiData (version 3.1) database. Regular reports will be produced on (1) patient accrual and follow-up completion/retention in relation to goals and timeline, (2) the randomization process and group comparability on the balancing variables, (3) key baseline characteristics of the sample, by (blinded) group, related to the primary and secondary outcome variables and proposed effect modifiers and mediators, (4) intervention exposure and adherence, and (5) protocol violations. Any observed delays in these processes or data irregularities will be followed up and resolved in a timely manner.

The complete de-identified dataset of the trial will be publicly available when finalized. Details of the data and safety monitoring plan and the study governance structure are shown in the Multimedia Appendix 4.

### Statistical Analyses

We will assess randomization across the two arms by comparing sociodemographic characteristics and other potential confounding variables using chi-square for categorical variables, and *t* tests or other equivalent nonparametric tests, as appropriate, for continuous variables. Primary outcomes will be the difference in follow-up proportions of participants who meet MINI criteria for major depression and difference in follow-up function scores of participants in the intervention and control arms 6 months after the end of treatment.

Secondary outcomes will include measures of self-esteem, posttraumatic stress symptoms, social support, stigma, alcohol use, ART adherence, viral load, number of disability days, asset possession indices, and cost-effectiveness data. Primary outcomes, secondary outcomes, and all subgroup analyses will be analyzed by ITT using a multilevel mixed model adjusting for clustering in health centers to determine effect modifiers [49]. The status of randomization (intervention/control) and baseline depression score will be included as a covariate. To account for the clustered design, we will treat clusters as a random effect. Potential confounding variables (eg, age, gender, socioeconomic status) with significant differences across intervention and control arms will be included as fixed factors. The R statistical software will be used to conduct all analyses [50]. A process evaluation using mixed methods to assess acceptability, feasibility, fidelity, causal mediating processes, and contextual influences in the trial will be conducted.

The primary analysis will follow ITT principles and use all available follow-up data, with missing data handled directly through maximum likelihood estimation in mixed modeling. We will document the extent and pattern of missing data and the reasons and will conduct sensitivity analyses of the impact of missing data on stability of the primary results. Missing values will be imputed with multiple imputations. We will verify that mixed model‒based results are not sensitive to violations of model assumptions with permutation tests.

Last, Cohen *d* effect sizes will be computed for the effect estimates to determine the size of the intervention effect [51].

For the qualitative data, interview transcripts will be reviewed for accuracy and then transcribed verbatim before translation into English. To control for errors in translation, two research assistants fluent in English and the local language (Luo) will work together to translate and electronically transcribe the data [52]. QRS NVivo 10 qualitative data analysis software will be used for coding and thematic analysis [53].

The interview data will initially be coded according to a number of themes that corresponded to the focus questions. The codes will be used to construct matrix displays based on the co-occurrence of codes and the two treatment groups. The resulting matrix display will provide both the frequency of responses and the detailed content of responses, allowing us to assess how often responses varied between the two treatment groups. Intercoder reliability will be assessed.

## Results

The trial has been approved by the Makerere College of Health Sciences School of Health Sciences Research Ethics Committee, TASO, and the Uganda National Council of Science and Technology. A Data and Safety Monitoring Board has been put in place to monitor trial progress. A total of 1140 persons living with HIV have been recruited to the trial. An analysis of baseline and 6-month data is in progress.

The results of this trial will not only be presented at national and international conferences but also submitted for publication in peer-reviewed journals and as a report to the funding agencies.

## Discussion

### Principle Findings

The GSP intervention is a complex intervention containing several interacting components, requires those delivering the intervention to acquire certain competencies and skills, and is associated with a variety of outcomes. Its development was prompted by the need to narrow the gap between the availability and need for depression treatment among HIV-positive populations in poor resource settings. The prior feasibility study [54] and pilot randomized clinical trial [36] of this intervention served to test these theories, procedures, recruitment, retention, and explore our hypothesized change processes and outcomes. The process evaluation data of this trial indicated that acquisition of knowledge and skills that enhance social connections, support, and better coping strategies with adverse situations leads to a reduction in depression symptoms. The absence of depression improves ability to work and obtain savings and other livelihood assets. The pursuit of livelihoods helps restore the dignity and independence that sustain a reduction in depression and improvement in functioning [37].

The Social, Emotional, and Economic empowerment through Knowledge of Group Support Psychotherapy (SEEK-GSP) trial will provide robust evidence for the change processes and outcomes we observed in the pilot studies. Further, the trial will demonstrate the potential for dissemination and integration into existing HIV service delivery platforms of a culturally sensitive first line treatment for mild to moderate depression. Depression poses a major burden on persons living with HIV/AIDS, particularly those in poor resource settings where poverty and mental health interact in a negative cycle [55]. Treating depression in PLWH is critical to the realization of the “90-90-90 targets” by 2020 aiming to diagnose 90% of all HIV positive people, provide ART for 90% of those diagnosed, and achieve viral suppression for 90% of those treated [56].

Prior trials of psychological therapies for depression in poor resource settings have shown that they result in significant reduction in depression symptoms and an increase in functioning levels [57,58]. However, both trials attracted mostly women. The SEEK-GSP trial specifically addresses this gap in evidence and in mental health services for a highly vulnerable HIV population in a primary care setting.

The SEEK-GSP trial targets both men and women living with HIV who have been exposed to decades of brutal civil war. Many of these individuals live in extreme poverty, with little to no education, in remote rural villages and rely on subsistence farming for survival. The vast majority have multiple experiences of war-related trauma and/or gender-based violence. This target group is a good example of what has been termed “intersecting populations”—groups that are vulnerable on multiple levels and disadvantaged across many determinants of health [59]. In this case, war survivors are made even more vulnerable by HIV/AIDS, unemployment, poverty, and food insecurity. Those living in remote villages with little access to services are even more vulnerable. This trial will demonstrate the extent to which primary care providers’ influence can be leveraged to motivate affected individuals to attend group sessions, manage issues that arise during engagement, and support practice of learned skills. Such primary care‒based interventions also provide health care system opportunities to support culturally appropriate services that minimize unintended negative impacts and maximize positive impacts for vulnerable groups.

### Limitations

Limitations of the SEEK-GSP trial relate to generalizability to other ethnic populations in Uganda. Also, the adherence measurement methods used in this study have limitations. However, to date there is no established gold standard to measure ART adherence. In the analyses of data from this trial, we shall assess the sensitivity of our adherence measure by determining whether it was able to predict viral suppression.

Despite these limitations, the trial will provide critical evidence to support culturally centered psychological therapy for depression in primary care settings. Trial outcomes will be supplemented with process evaluation outcomes that will provide contextual information that health care and public health stakeholders can use to guide implementation decisions for their particular setting [60].

### Conclusions

Confirmation of our primary hypothesis and supportive secondary data can critically inform the national dissemination and implementation of GSP to treat mild to moderate depression in PLWH. Results may provide greater insight into the partnerships between LHWs and existing government health systems in low-resource settings and may lead to policy formulation regarding the integration of GSP into HIV care services on a country-wide basis.

## Appendix

Multimedia Appendix 1 SPIRIT 2013 Checklist.

Multimedia Appendix 2 The content of group support psychotherapy and group HIV education group sessions.

Multimedia Appendix 3 Group support psychotherapy core competencies and subcompetencies.

Multimedia Appendix 4 Data safety and management plan.

## Abbreviations

AIDS: acquired immune deficiency syndrome
ART: antiretroviral therapy
GHE: group HIV education
GSP: group support psychotherapy
ITT: intention-to-treat
LHW: lay health worker
MINI: Mini International Neuropsychiatric Interview
PHC: primary care health center
PLWH: person living with HIV
SEEK-GSP: Social, Emotional, and Economic empowerment through Knowledge of Group Support Psychotherapy
TASO: The AIDS Support Organization
WHO: World Health Organization
