# Supplementary material for: The Effect of Group Support Psychotherapy Delivered by Trained Lay Health Workers for Depression Treatment Among People with HIV in Uganda: Protocol of a Pragmatic, Cluster Randomized Trial
Source: JMIR Res Protoc. 2017 Dec 11;6(12):e250. doi: 10.2196/resprot.8925 (PMC5742658; doi:10.2196/resprot.8925)
Supplement: Multimedia Appendix 2 [file resprot_v6i12e250_app2.pdf]

	Group Support Psychotherapy	Group HIV education
**Session 1**	Group facilitators (GFs) assist in building group cohesion, explain the therapeutic process of GSP, guide the group members (GMs) as they lay the ground rules and elect a group leader among them. GFs explained how GSP will heal depression and reassure GMs that the GSP intervention will provide a safe and supportive environment in which confidentiality is maintained. GMs are asked to make a commitment to attend all group sessions.	The first session of GHE focuses on introductory issues, building group cohesion, explaining the rationale of HIV education. GFs guide GMs as they lay the ground rules and elect a group leader among them.
**Session 2**	The GFs educate GMs about depression and its relationship with HIV/AIDS, the presentation, triggers, complications and treatment options for depression and help GMs understand the link between HIV and depression. The impact of depression on adherence to antiretroviral medications is emphasized in this session.	In the second session, GFs educate about the progression of HIV in the body. Emphasis is placed on explaining the four stages of HIV infection. Also, the difference between HIV and AIDS is explained
**Session 3&4**	GFs encourage GMs to share their personal problems with others, to seek support and to receive and provide feedback. The GF engages the group in a discussion on the pros and cons of coping strategies to deal with their personal problems.	In sessions 3 and 4, the GFs educates about the various ways of transmission and prevention of HIV infection.
**Session 5**	GFs highlight the depressive thinking and excessive worries that GMs demonstrated as they shared their personal problems. GFs teach GMs how to identify positive and negative ways of thinking and how to replace negative thoughts with positive thoughts. GFs also demonstrate how to positively cope with excessive worries usually experienced when one is going through tough situations.	In sessions 5 and 6, the GFs educate about mother-to-child transmission of HIV and explain specific ways in which the HIV virus may be transmitted from a mother to her child.
**Session 6**	GFs help group members learn how to solve problems and how to cope with stigma at personal, family, and community level.	
**Session 7&8**	In session 7, GFs illustrate basic livelihood skills that will enable GMs to identify income generating activities that will improve their livelihoods thus enabling them to take control of their lives.<br>In session 8, GMs are asked to demonstrate basic livelihood skills learned by allowing them to present their business plans to the group. Discussions are held about various business ideas presented by the GMs.	The last two sessions focused on basic facts about antiretroviral therapy (ART) with emphasis on side effects, drug interactions and the importance of ART adherence to prevent emergence of drug resistance.
