# Supplementary material for: The Effect of Group Support Psychotherapy Delivered by Trained Lay Health Workers for Depression Treatment Among People with HIV in Uganda: Protocol of a Pragmatic, Cluster Randomized Trial
Source: JMIR Res Protoc. 2017 Dec 11;6(12):e250. doi: 10.2196/resprot.8925 (PMC5742658; doi:10.2196/resprot.8925)
Supplement: Multimedia Appendix 3 [file resprot_v6i12e250_app3.pdf]

**Competency Domain**	**Knowledge**	**Skills**	**Attitudes**
Able to initiate contact and build rapport with a depressed PLWH	Describes basic communication skills required for effective counseling	Applies principles of active and reflective listening skills, expressing empathy and establishing rapport	Expresses a positive manner of relating to others by being warm and nonjudgmental, and showing respect for people and their differences (e.g. ethnic, age-related, or political)
Able to conduct brief assessment and triage	Defines characteristics of mild-moderate versus severe depression	Gives health talks on depression and performs screening to distinguish between mild-moderate versus severe depression	Reports that the screening is useful to discern between mild-moderate versus severe depression
Able to deliver GSP following the Intervention Manual	Describes the importance of mitigating depression and enhancing positive coping and income generating abilities	Demonstrates techniques for mitigating depression and enhancing positive coping and income generating abilities	Expresses confidence in ability to mitigate suicidal ideation and promote positive coping, problem solving, and income generating skills.
Able to practice self-awareness and self-care (a prerequisite for caring for others)	Identifies at least 5 possible signs of personal stress, and knows at least 5 self-care principles and practices such as proper nutrition, exercise and sleep hygiene	Applies appropriate techniques for maintaining awareness of possible signs of personal stress, and for using self-care principles.	Able to stay calm and confidently address strong release of emotions during group sessions.<br>Able to manage personal stress, by using self-care principles.
