# Supplementary material for: The Effect of Group Support Psychotherapy Delivered by Trained Lay Health Workers for Depression Treatment Among People with HIV in Uganda: Protocol of a Pragmatic, Cluster Randomized Trial
Source: JMIR Res Protoc. 2017 Dec 11;6(12):e250. doi: 10.2196/resprot.8925 (PMC5742658; doi:10.2196/resprot.8925)
Supplement: Multimedia Appendix 4 [file resprot_v6i12e250_app4.pdf]

Oversight responsibilities: This trial is a low risk study given that the therapy under investigation is non-evasive and is targeting mild to moderate depression. Oversight of the trial is provided by the Principal Investigator (PI) and co-investigators who is actively involved in the conduct of the study throughout assisted by study coordinators –two in each of the three participating districts.

Monitoring procedures: The PI ensured that informed consent was obtained prior to performing any research procedures, that all subjects meet eligibility criteria, and that the study was conducted according to the IRB-approved research plan. Study data is accessible at all times to the PI and co-investigators to review.

The PI has constituted a data and safety monitoring board (DSMB) consisting of a Professor of Epidemiology, Professor of Bio-statistician, and an Associate Professor of HIV Psychiatry to assist in the independent review of data, in particular, data concerning accrual, drop-outs, protocol deviations and adverse events. The PI and co-investigators review adverse effects (AEs) individually in real-time and in aggregate on a monthly basis. The PI and co-investigators review serious adverse events (SAEs), such as sudden deaths, attempted suicide in real-time and this is entered into the SAE forms and submitted to both the DSMB the research ethics committee for review. The PI ensures all protocol deviations, AEs, and SAEs are reported to the DSMB and relevant IRBs according to the applicable regulatory requirements.

Collection and Reporting of SAES and AES: For this study, the following standard AE definitions are used:

Adverse event: Any unfavorable and unintended sign or symptom associated with the participation in group support psychotherapy, regardless of whether it is considered related to the therapy. **Serious Adverse Event:** Any AE that results in any of the following outcomes:

Death, Life-threatening, Event requiring inpatient hospitalization, Persistent or significant disability/incapacity. AEs are graded according to the following scale

Mild: An experience that is transient, & requires no special treatment or intervention. The experience does not generally interfere with usual daily activities. **Moderate:** An experience that is alleviated with simple therapeutic treatments. The experience impacts usual daily activities. **Severe:** An experience that requires therapeutic intervention. The experience interrupts usual daily activities. If hospitalization (or prolongation of hospitalization) is required for treatment it becomes an SAE.

The study uses the following AE attribution scale:

Not related: The AE is clearly not related to the study procedures (i.e., another cause of the event is most plausible and/or a clinically plausible temporal sequence is inconsistent with the onset of the event). **Possibly related:** An event that follows a reasonable temporal sequence from the initiation of study procedures, but that could readily have been produced by a number of other factors. **Related:** The AE is clearly related to the study procedures.

During participant recruitment, all participants received a suicide risk assessment and those with high suicide risk were not eligible to participate in the study. Group facilitators were asked to check in on participant's feelings and thoughts at the start of each group session with the aim of eliciting suicidal thoughts. Any suicidal thoughts were addressed in the group discussion. If a group member did not return for a session, a group member was tasked to check on the patient and report on his or her condition in the next meeting. SAEs and specific therapy-associated AEs are reported to the DSMB and the School of Health Sciences Research and Ethics Committee which is overseeing the study.

Management of Risks to Subjects

Expected AEs associated with this study would be suicide attempts due to lack of treatment response to the group support psychotherapy. During participant recruitment, all participants received a suicide risk assessment (Patterson et al's 1983 *Evaluation of suicidal patients: the SAD PERSONS scale*). Individuals with depression with high suicide risk were excluded from the study. Those with a low to moderate risk were included, their thoughts were assessed at every group meeting and care givers were asked to keep close watch on the affected individual. If suicidal thoughts were still present after 4 sessions of GSP or GHE, these individuals were referred to a mental health worker.

STOPPING RULES: Persistent suicide thoughts or attempt is the main adverse event and safety issue in the study. Previous studies that 15-20% of patients with depression commit suicide. The incidence of persistent suicide thoughts or attempt will be compared across treatment arms that is: Number of patients referred because of persistent suicide thoughts within 90-days of randomization and number of suicide attempts occurring within 90-days of randomization. If there is a significant difference between the two groups, the trial will be stopped at that point and all patents in the control group will receive group support psychotherapy. If there are no significant differences the trial continues until 6 months after the end of group support psychotherapy sessions.

Study governance structure

The Ministry of Health, Mental health program, The AIDS Support Organization (TASO), and Makerere University, Department of Psychiatry formally agreed to work in partnership for the purpose of implementing the SEEK-GSP trial. They signed a Memorandum of Understanding (MOU) which defined the goals, objectives and respective responsibilities of each party. The table below summarizes the roles of the collaborating institutions. A steering

committee consisting of the principle investigator and co-investigators based in Uganda has been responsible for implementing and supervising research activities. This committee has been supported by a technical advisory committee consisting of international co-investigators who have given technical advice on research design, analytic and dissemination strategy based on areas of expertise of the members of the technical advisory committee.

An independent Data and Safety Monitoring Board (DSMB) has been established by the Steering Committee, composed a senior biostatistician, an expert in HIV and Mental health and a senior epidemiologist with vast experience in conduct of clinical trials, none of whom are involved with the study. Members of the DSMB have signed an agreement with the Principle investigator which specifies terms of reference. The DSMB will assess the performance of overall study operations and any other relevant issues, including 1) Interim/cumulative data for evidence of study-related adverse events; 2) Interim/cumulative data for evidence of efficacy according to pre-established statistical guidelines, if appropriate; 3) Data quality, completeness, and timeliness; 4) Performance of individual centers; 5) Adequacy of compliance with goals for recruitment and retention; 6) Adherence to the protocol; and 7) Factors that might affect the study outcome (such as protocol violations, etc.)

ROLES OF COLLABORATING INSTITUTIONS

Stage of model implementation	Makerere, Department of Psychiatry	Ministry of Health , Mental Health Program	The AIDS Support Organization(TASO)
Pre-intervention	• Develop the project proposal<br>• Obtain funding<br>• Provide overall guidance in the planning and implementation of the project.<br>• Conduct community sensitization meetings and oversee formation of community advisory board<br>• Conduct baseline evaluations including training needs assessment<br>• Obtain IRB approvals	• Collaborate with Makerere and TASO in designing training materials for professional and lay health workers.<br>• Collaborate with Makerere to identify facilitators of training sessions.<br>• Collaborate with Makerere to identify supervisors who will supervise the trained HIV care	• Identify TASO HIV care providers to be trained in delivery of GSP<br>• Identify lay health workers involved in HIV care to be trained in delivery of GSP<br>• TASO Gulu HIV care providers will link the project team to lay health

	• Registration of Trial<br>• Collaborate with TASO to identify TASO HIV care providers trained in delivery of GSP<br>• Collaborate with TASO to identify lay health workers involved in HIV care to be trained in delivery of GSP<br>• Collaborate with MOH to designing training materials for professional and lay health workers.	providers as they conduct trainings for lay health workers.	workers involved in HIV care who are targets of our training program.<br>• TASO Gulu team will participate in the training and supervision of the LHW training in GHE delivery.
Intervention	• Provide specialists to train HIV care providers in screening for depression, provision of first line treatment –GSP and referral of non-responsive cases.<br>• Conduct training evaluations, as appropriate<br>• Collaborate with MOH in supervision of training workshops for lay health workers.<br>• Provide research assistants to conduct follow up assessments	• Organize and oversee all training workshops in which HIV primary health care workers and TASO HIV care providers will be trained to recognize and treat mild-moderate depression with Group Support Psychotherapy.<br>• Supervise trained primary health workers as they train lay health workers to recognize and treat mild-moderate depression with Group Support Psychotherapy in the villages.<br>• Organize workshops with stakeholders to develop policy guidelines for the integration of depression treatment with Group Support Psychotherapy into HIV service delivery in Uganda.	• Selected TASO HIV care providers will attend training sessions.<br>• Trained TASO HIV care providers will then train selected lay health workers<br>• Trained TASO HIV care providers will supervise lay health workers as they deliver GSP
Post-intervention	• Supervise follow-up assessments of both trainees and study participants.<br>• Analysis of project data<br>• Writing manuscripts	• Lead policy development activities	• Participate in process evaluation & follow up assessments.<br>• Work with partners to continue training of other HIV care providers in other TASO branches in screening for depression and offer first line treatment - GSP.
